# Genomic best linear unbiased prediction method including imprinting effects for genomic evaluation

**DOI:** 10.1186/s12711-015-0091-y

**Published:** 2015-04-19

**Authors:** Motohide Nishio, Masahiro Satoh

**Affiliations:** NARO Institute of Livestock and Grassland Science, 305-0901 Ikenodai 2, Tsukuba, Japan

## Abstract

**Background:**

Genomic best linear unbiased prediction (GBLUP) is a statistical method used to predict breeding values using single nucleotide polymorphisms for selection in animal and plant breeding. Genetic effects are often modeled as additively acting marker allele effects. However, the actual mode of biological action can differ from this assumption. Many livestock traits exhibit genomic imprinting, which may substantially contribute to the total genetic variation of quantitative traits. Here, we present two statistical models of GBLUP including imprinting effects (GBLUP-I) on the basis of genotypic values (GBLUP-I1) and gametic values (GBLUP-I2). The performance of these models for the estimation of variance components and prediction of genetic values across a range of genetic variations was evaluated in simulations.

**Results:**

Estimates of total genetic variances and residual variances with GBLUP-I1 and GBLUP-I2 were close to the true values and the regression coefficients of total genetic values on their estimates were close to 1. Accuracies of estimated total genetic values in both GBLUP-I methods increased with increasing degree of imprinting and broad-sense heritability. When the imprinting variances were equal to 1.4% to 6.0% of the phenotypic variances, the accuracies of estimated total genetic values with GBLUP-I1 exceeded those with GBLUP by 1.4% to 7.8%. In comparison with GBLUP-I1, the superiority of GBLUP-I2 over GBLUP depended strongly on degree of imprinting and difference in genetic values between paternal and maternal alleles. When paternal and maternal alleles were predicted (phasing accuracy was equal to 0.979), accuracies of the estimated total genetic values in GBLUP-I1 and GBLUP-I2 were 1.7% and 1.2% lower than when paternal and maternal alleles were known.

**Conclusions:**

This simulation study shows that GBLUP-I1 and GBLUP-I2 can accurately estimate total genetic variance and perform well for the prediction of total genetic values. GBLUP-I1 is preferred for genomic evaluation, while GBLUP-I2 is preferred when the imprinting effects are large, and the genetic effects differ substantially between sexes.

## Background

Genomic imprinting is an epigenetic process that involves DNA methylation and histone modifications that distinguish the expression of maternal and paternal alleles [1]. The expression of an imprinted gene depends on the parent from which it is inherited. Complete inactivation of an imprinted gene results in functional haploidy, with only one of the two copies of the gene expressed. Well known examples of such imprinted genes are *IGF2* (*insulin-like growth factor 2*) in pigs [2] and the Callipyge gene in sheep [3]. Moreover, imprinting may not entail the complete inactivation of a gene. In a study of peripheral blood leukocytes in humans, four of 38 cases exhibited substantial biallelic expression of *IGF2*, although the product level of the maternally-derived gene was lower than that of the paternally-derived gene in all cases [4]. Over 70 imprinted genes have been identified in mice [5], and 24 genes with parent-of-origin effects in beef cattle [6]. Furthermore, quantitative traits such as carcass composition, growth, teat number, and litter size have been suggested to exhibit imprinting effects [7-10]. Thus, imprinting effects may substantially contribute to the total genetic variation of quantitative traits.

There are several statistical methods for modeling imprinting effects. Using a mixed model, Schaeffer et al. [11] replaced the numerator relationship matrix with a gametic relationship matrix to calculate the expectation of covariance among relatives with imprinting. Essl and Voith [12] suggested that sire and dam models should be constructed separately to assess differences between paternal and maternal imprinting. Neugebauer et al. [13,14] recently fitted a model with correlated paternal and maternal gametes to simultaneously estimate imprinting variances between sexes in pigs and beef cattle. These methods are based on the traditional best linear unbiased prediction (BLUP) method, which uses only pedigree information. More recently, the genomic BLUP (GBLUP) method was developed by modifying the BLUP method to incorporate single nucleotide polymorphism (SNP) information in the form of a genomic relationship matrix that defines the additive genetic covariance among individuals. GBLUP includes genomic information into breeding value estimation and has been used for genomic selection in dairy cattle [15-18]. Therefore, modeling genetic effects by including imprinting effects is expected to improve the predictive ability of GBLUP. Thus, the objectives of this study were twofold: (1) develop a GBLUP method including imprinting effects (termed GBLUP-I hereafter) and (2) estimate genetic variances and assess the accuracies and unbiasedness of genomic predictions using simulation data with varying degrees of imprinting.

## Methods

### Genetic model

Spencer [19] first extended the standard two-allele one locus model of quantitative genetics to account for imprinting. Following the approach of Spencer [19], consider an autosomal biallelic locus with alleles *A*_1_ and *A*_2_ at frequencies 1−*q* and *q*, respectively, in the population. Allele frequencies of males and females were assumed to be the same and under Hardy-Weinberg equilibrium. By denoting a genotype, *A*_*i*_*A*_*j*_, *A*_*i*_ and *A*_*j*_, are the paternally- and maternally-derived alleles, respectively. Following the approach of Spencer [19], the genotypic values for genotypes *A*_1_*A*_1_, *A*_1_*A*_2_, *A*_2_*A*_1_, and *A*_2_*A*_2_ are then given by *a*, *d*_1_, *d*_2_, and -*a*, respectively. In this study, the mean of two heterozygotes and the difference between two heterozygotes were defined as *δ* and *ε*:

$$ \delta =\frac{d_1+{d}_2}{2} $$

and

$$ \varepsilon =\frac{d_1-{d}_2}{2} $$

**Figure 1:**
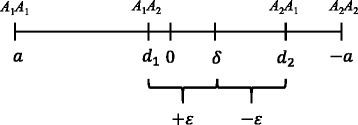
**Genotypic values for the four genotypes (** ***A***_1_ ***A***_1_ **,** ***A***_1_ ***A***_2,_ ***A***_2_ ***A***_1, and_ ***A***_2_ ***A***_2_ **).**

In this model, the heterozygous genotypic values were + *ε* and −*ε* deviations from *δ*, i.e., *d*_1_ and *d*_2_ can be rewritten as *δ* + *ε* and *δ* − *ε*, respectively (Figure 1). With imprinting, reciprocal heterozygotes differ in their genotypes. For example, in the case of complete inactivation of the maternal allele (i.e., *ε* = *a* and *δ* = 0), the genotypic value of *A*_2_*A*_1_ is the same as that of *A*_2_*A*_2_, whereas the genotypic value of *A*_1_*A*_2_ is the same as that of *A*_1_*A*_1_. If the paternally-derived *A*_1_ allele randomly combines with maternally-derived alleles from a population, the frequencies of the genotypes produced will be 1−*q* for *A*_1_*A*_1_ and *q* for *A*_1_*A*_2_. The genotypic values of *A*_1_*A*_1_ and *A*_1_*A*_2_ are *a* and *d*_1_, respectively. Taking in account the proportions at which they occur, the mean value of genotypes produced from the paternally-derived *A*_1_ allele is (1−*q*)*a + qd*_1_. The mean genotypic value in the entire population (*μ*) is as follows:

$$ \begin{array}{c}\hfill \mu ={\left(1-q\right)}^2\cdot a+\left(1-q\right)q\cdot {d}_1+\left(1-q\right)q\cdot {d}_2+{q}^2\cdot \left(-a\right)\hfill \\ {}\hfill =\left(1-2q\right)a+2\left(1-q\right)q\delta .\hfill \end{array} $$

Thus, the average effect of the paternally-derived *A*_1_ allele is calculated from the difference between the mean value of the genotypes produced and population mean as follows:

$$ \begin{array}{l}\left(1-q\right)a+q{d}_1-\left\{\left(1-2q\right)a+2\left(1-q\right)q\delta \right\}\\ {}=q\left\{a+\left(2q-1\right)\delta +\varepsilon \right\}=q{\alpha}_m,\end{array} $$

where *α*_*m*_ is the average effect of the allele substitution in the paternal gamete and is equivalent to the male breeding value of Spencer [19]. Similarly, the average effect of the maternally-derived *A*_1_ allele is as follows:

$$ q\left\{a+\left(2q-1\right)\delta -\varepsilon \right\}=q{\alpha}_f, $$

where *α*_*f*_ is the average effect of the allele substitution in the maternal gamete and is equivalent to the female breeding values of Spencer [19]. The average effects of all alleles are in Table 1.

**Table 1:** **Average effects of paternal and maternal alleles at a QTL with imprinting**

**Gamete type**	**Allele**	**Values and frequencies of genotypes produced**				**Mean value of genotypes produced**	**Average allele effect**
		***A***_1_ ***A***_1_	***A***_1_ ***A***_2_	***A***_2_ ***A***_1_	***A***_2_ ***A***_2_		
		***a***	***δ+ε***	***δ-ε***	***-a***		
Sire	*A*_1_	1-*q*	*q*			(1-*q*)*a + q*(*δ+ε*)	*q*{*a +* (2*q*-1)*δ+ε*} = *qα*_*m*_
	*A*_2_			1-*q*	*q*	*-qa +* (1-*q*)(*δ-ε*)	-(1-*q*){*a +* (2*q*-1)*δ+ε*} = -(1-*q*)*α*_*m*_
Dam	*A*_1_	1-*q*		*q*		(1-*q*)*a + q*(*δ-ε*)	*q*{*a +* (2*q*-1)*δ-ε*} = *qα*_*f*_
	*A*_2_		1-*q*		*q*	*-qa +* (1-*q*)(*δ+ε*)	-(1-*q*){*a +* (2*q*-1)*δ-ε*} = -(1-*q*)*α*_*f*_

*α*_*m*_ = *a* + (2*q* - 1)*δ* + *ε*; *α*_*f*_ = *a* + (2*q* - 1)*δ* − *ε*; *a* = genotypic value of *A*_1_ *A*_1_; *δ* = mean of two heterozygotes; *ε* = difference between two heterozygotes; *q* = frequency of allele *A*_2_.

The genotypic deviation of a particular genotype can be calculated from the difference between its genotypic value and the population mean. For example, the genotypic deviation of *A*_1_*A*_2_ is as follows:

$$ \begin{array}{c}\hfill {d}_1-\mu =\left(2q-1\right)a+\left\{1-2\left(1-q\right)q\right\}\delta +\varepsilon \hfill \\ {}\hfill =\left(2q-1\right)\alpha +2\left(1-q\right)q\delta +\varepsilon, \hfill \end{array} $$

where *α = a +* (*2q −* 1)*δ* When there is no imprinting (*d*_1_ *= d*_2_ = *δ* and *ε =* 0) *α* is the same as the average effect of the allele substitution [20] and (2*q* − 1)*α* and 2(1 − *q*)*qδ* are the same as the breeding value and dominance deviation of the traditional genetic model. By using *δ* and *ε*, a genotypic deviation can be divided into three terms. Under imprinting, the breeding values and dominance deviations are no longer uncorrelated, which means that the total genetic variance cannot be partitioned into the usual additive and dominance variance [19]. Therefore, in this study, total genetic variance was partitioned into three variances corresponding to *α, δ*, and *ε*$\left({\sigma}_{a\hbox{'}}^2,{\sigma}_{d\hbox{'}}^2,\mathrm{and}\;{\sigma}_{i^{\hbox{'}}}^2\right)$ as follows:

$$ \begin{array}{c}\hfill {\sigma}_g^2=2\left(1-q\right)q{\alpha}^2+{\left\{2q\left(1-q\right)\right\}}^2{\delta}^2+2\left(1-q\right)q{\varepsilon}^2\hfill \\ {}\hfill ={\sigma}_{a^{\hbox{'}}}^2+{\sigma}_{d^{\hbox{'}}}^2+{\sigma}_{i\hbox{'}}^2.\hfill \end{array} $$

When there is no imprinting, ${\sigma}_{a\hbox{'}}^2$ and ${\sigma}_{d\hbox{'}}^2$ are the same as the additive and dominance genetic variance, respectively. In this case, the covariance between the *α* and *δ* terms (*σ*_*a* ' __*d* '_) is equal to 0, as follows:

$$ \begin{array}{l}{\sigma}_{a^{\hbox{'}}{d}^{\hbox{'}}}={\left(1-q\right)}^2\cdot 2q\alpha \cdot \left(-2{q}^2\delta \right)\\ {}+\left(1-q\right)q\cdot \left(2q-1\right)\alpha \cdot 2\left(1-q\right)q\delta \\ {}+\left(1-q\right)q\cdot \left(2q-1\right)\alpha \cdot 2\left(1-q\right)q\delta \\ {}+{q}^2\cdot -2\left(1-q\right)\alpha \cdot \left\{-2{\left(1-q\right)}^2\delta \right\}=0.\end{array} $$

The covariance between the *α* and *ε* terms (*σ*_*a* ' *i* '_) is also equal to 0, as follows:

$$ \begin{array}{l}{\sigma}_{a^{\hbox{'}}{i}^{\hbox{'}}}={\left(1-q\right)}^2\cdot 2q\alpha \cdot 0\\ {}+\left(1-q\right)q\cdot \left(2q-1\right)\alpha \cdot \varepsilon \\ {}+\left(1-q\right)q\cdot \left(2q-1\right)\alpha \cdot \left(-\varepsilon \right)\\ {}+{q}^2\cdot -2\left(1-q\right)\alpha \cdot 0=0.\end{array} $$

Similarly, the covariance between the *δ* and *ε* terms is also equal to 0.

Alternatively, paternal and maternal gametic variances (${\sigma}_{p_{at}}^2$ and ${\sigma}_{m_{at}}^2$, respectively) can be calculated from the variances of the average effects of paternally- and maternally-derived alleles:

$$ \begin{array}{c}\hfill {\sigma}_{p_{at}}^2=\left(1-q\right)\cdot {\left(q{\alpha}_m\right)}^2+q\cdot {\left\{-\left(1-q\right){\alpha}_m\right\}}^2\hfill \\ {}\hfill =\left(1-q\right)q{\alpha}_m^2,\hfill \end{array} $$

and

$$ \begin{array}{c}\hfill {\sigma}_{m_{at}}^2=\left(1-q\right)\cdot {\left(q{\alpha}_f\right)}^2+q\cdot {\left\{-\left(1-q\right){\alpha}_f\right\}}^2\hfill \\ {}\hfill =\left(1-q\right)q{\alpha}_f^2.\hfill \end{array} $$

The sum of these variances is as follows:

$$ \begin{array}{l}{\sigma}_{p_{at}}^2+{\sigma}_{m_{at}}^2=pq{\alpha}_m^2+pq{\alpha}_f^2\\ {}=2\left(1-q\right)q{\left\{a+\left(2q-1\right)\delta \right\}}^2+2\left(1-q\right)q{\varepsilon}^2\\ {}={\sigma}_{a\hbox{'}}^2 + {\sigma}_{i\hbox{'}}^2.\end{array} $$

Thus, the total genetic variance can be partitioned as follows:

$$ {\sigma}_g^2={\sigma}_{p_{at}}^2+{\sigma}_{m_{at}}^2+{\sigma}_{d\hbox{'}}^2. $$

### Statistical model

Two statistical models of GBLUP-I based on genotypic values (GBLUP-I1) and gametic values (GBLUP-I2) are proposed here.

First, GBLUP-I1 is defined as follows:

$$ \mathbf{y}=\mathbf{X}\boldsymbol{\upbeta } +{\mathbf{Z}}_{\mathbf{a}}\mathbf{a}+{\mathbf{Z}}_{\mathbf{d}}\mathbf{d}+{\mathbf{Z}}_{\mathbf{i}}\mathbf{i}+\mathbf{e}, $$

where **y** is the vector of the phenotypes; **β** is the vector of the fixed effects; **a**, **d**, and **i** are the vectors of *α*, *δ*, and *ε* terms, respectively; **X**, **Z**_**a**_, **Z**_**d**_, and **Z**_**i**_ are incidence matrices linking the phenotypes to **β**, **a**, **d**, and **i**, respectively; and **e** is the vector of errors. The variances of **a**, **d**, and **i** are as follows:

$$ \mathrm{V}\mathrm{a}\mathrm{r}\left(\mathbf{a}\right)={\mathbf{G}}_{\mathbf{a}}{\sigma}_{a\hbox{'}}^2, $$

$$ \mathrm{V}\mathrm{a}\mathrm{r}\left(\mathbf{d}\right)={\mathbf{G}}_{\mathbf{d}}{\sigma}_{d\hbox{'}}^2, $$

and

$$ \mathrm{V}\mathrm{a}\mathrm{r}\left(\mathbf{i}\right)={\mathbf{G}}_{\mathbf{i}}{\sigma}_{i\hbox{'}}^2, $$

where **G**_**a**_, **G**_**d**_, and **G**_**i**_ are the genomic relationship matrices relevant to *α*, *δ*, and *ε* terms, respectively. These matrices describe the relationships among genotyped individuals and can be constructed by using the information from genome-wide SNPs. Let *A*_*1j*_ and *A*_*2j*_ be two alleles at the *j*^th^ SNP and *q*_*j*_ be the frequency of *A*_2*j*_. **G**_**a**_ and **G**_**d**_ are the same as the genomic relationship matrices for breeding values and dominance deviations without imprinting. Thus, **G**_**a**_ and **G**_**d**_ can be calculated as described previously [21,22]:

$$ {\mathbf{G}}_{\mathbf{a}}=\frac{{\mathbf{M}}_{\mathbf{a}}{{\mathbf{M}}_{\mathbf{a}}}^{\hbox{'}}}{{\displaystyle {\sum}_j^{N_{snp}}}2{q}_j\left(1-{q}_j\right)}, $$

and

$$ {\mathbf{G}}_{\mathbf{d}}=\frac{{\mathbf{M}}_{\mathbf{d}}{\mathbf{M}}_{\mathbf{d}}^{\hbox{'}}}{{\displaystyle {\sum}_j^{N_{snp}}}{\left\{2{q}_j\left(1-{q}_j\right)\right\}}^2}, $$

where **M**_**a**_ and **M**_**d**_ are *n* × *N*_*snp*_ matrices (*n* is the number of genotyped individuals, and *N*_*snp*_ is the number of SNPs); the elements of **M**_**a**_ and **M**_**d**_ for the *i*^*th*^ individual at the *j*^*th*^ SNP are calculated as follows:

$$ {\mathbf{M}}_{\mathbf{a}\ i,j} = \left\{\begin{array}{l}2{q}_j\ \left({A}_1{A}_1\right)\hfill \\ {}2{q}_j-1\ \left({A}_1{A}_2\ \mathrm{and}\ {A}_2{A}_1\right)\hfill \\ {}2{q}_j-2\ \left({A}_2{A}_2\right)\hfill \end{array}\right., $$

and

$$ {\mathbf{M}}_{\mathbf{d}\ i,j}=\left\{\begin{array}{l}-2{q}_j^2\ \left({A}_1{A}_1\right)\hfill \\ {}2{q}_j\left(1-{q}_j\right)\ \left({A}_1{A}_2\ \mathrm{and}\ {A}_2{A}_1\right)\hfill \\ {}-2{\left(1-{q}_j\right)}^2\ \left({A}_2{A}_2\right)\hfill \end{array}\right.. $$

Similarly, **M**_**i**_ is assumed to be a *n* × *N*_*snp*_ matrix, and the element of **M**_**i**_ for the *i*^th^ individual at the *j*^th^ SNP can be calculated as follows:

$$ {\mathbf{M}}_{\mathbf{i},i,j}=\left\{\begin{array}{r}\hfill 0\ \left({A}_1{A}_1\right)\\ {}\hfill 1\ \left({A}_1{A}_2\right)\\ {}\hfill -1\ \left({A}_2{A}_1\right)\\ {}\hfill 0\ \left({A}_2{A}_2\right)\end{array}\right. $$

The elements of **M**_**a**_, **M**_**d**_, and **M**_**i**_ describe the coefficients of the *α*, *δ*, and *ε*. terms in Table 2, respectively. Therefore, **i** and its variance can be derived as follows:

**Table 2:** **Genotypic values in the two-allele model**

	***A***_**1**_ ***A***_**1**_	***A***_**1**_ ***A***_**2**_	***A***_**2**_ ***A***_**1**_	***A***_**2**_ ***A***_**2**_
Genotypic value	*a*	$\delta$+$\varepsilon$	$\delta$-$\varepsilon$	*-a*
Deviation from population mean	2*qa*-2(1-*q*)*q* $\delta$	(2*q*-1)*a* + {1-2(1-*q*)*q*}$\delta$+$\varepsilon$	(2*q*-1)*a* + {1-2(1-*q*)}*q* $\delta$-$\varepsilon$	-2(1-*q*)*a-*2(1-*q*)*q* $\delta$
*α* term	2*qα*	(2*q*-1)*α*	(2*q*-1)*α*	-2(1-*q*)*α*
$\delta$ term	-2*q* ^2^ $\delta$	2(1-*q*)*q* $\delta$	2(1-*q*)*q* $\delta$	-2(1-*q*)^2^ $\delta$
$\varepsilon$ term	0	$\varepsilon$	-$\varepsilon$	0

*α* = *α* + (2*q*-1)$\delta$; *a* = genotypic value of *A*_1_ *A*_1_; $\delta$ = mean of two heterozygotes; $\varepsilon$ = difference between two heterozygotes; *q* = frequency of allele *A*_2_.

$$ \mathbf{i}={\mathbf{M}}_{\mathbf{i}}\boldsymbol{\varepsilon}, $$

where **ε** is the *N*_*snp*_ dimensional vector of which the *j*^*th*^ element is *ε*_*j*_. Thus, the variance of **i** is calculated as follows:

$$ \mathrm{V}\mathrm{a}\mathrm{r}\left(\mathbf{i}\right)={\mathbf{M}}_{\mathbf{i}}{\mathbf{M}}_{\mathbf{i}}^{\hbox{'}}\mathrm{V}\mathrm{a}\mathrm{r}\left(\varepsilon \right), $$

$$ {\sigma}_{i\hbox{'}}^2={\displaystyle \sum_j^{N_{snp}}}2{q}_j\left(1-{q}_j\right)\mathrm{V}\mathrm{a}\mathrm{r}\left(\varepsilon \right). $$

Consequently, **G**_**i**_ can be calculated using **M**_***i***_:

$$ {\mathbf{G}}_{\mathbf{i}}=\frac{{\mathbf{M}}_{\mathbf{i}}{\mathbf{M}}_{\mathbf{i}}^{\hbox{'}}}{{\displaystyle {\sum}_j^{N_{snp}}}2{q}_j\left(1-{q}_j\right)}. $$

In general, GBLUP includes only breeding values. The statistical model of GBLUP is as follows:

$$ \mathbf{y}=\mathbf{X}\boldsymbol{\upbeta } +{\mathbf{Z}}_{\mathbf{a}}\mathbf{a}+\mathbf{e}. $$

Therefore, without imprinting and dominance, the GBLUP model is the same as GBLUP-I1.

Second, GBLUP-I2 is defined as:

$$ \mathbf{y} = \mathbf{X}\boldsymbol{\upbeta } +{\mathbf{Z}}_{{\mathbf{p}}_{\mathbf{at}}}{\mathbf{p}}_{\mathbf{at}}+{\mathbf{Z}}_{{\mathbf{m}}_{\mathbf{at}}}{\mathbf{m}}_{\mathbf{at}}+{\mathbf{Z}}_{\mathbf{d}}\mathbf{d}+\mathbf{e}, $$

where **p**_**at**_ and **m**_**at**_ are the vectors of paternal and maternal gametic effects, respectively; and ${\mathbf{Z}}_{{\mathbf{p}}_{\mathbf{at}}}$ and ${\mathbf{Z}}_{{\mathbf{m}}_{\mathbf{at}}}$ are incidence matrices linking phenotypes to **p**_**at**_ and **m**_**at**_, respectively. The variances of **p**_**at**_ and **m**_**at**_ are as follows:

$$ \mathrm{V}\mathrm{a}\mathrm{r}\left({\mathbf{p}}_{\mathbf{at}}\right)={\mathbf{G}}_{{\mathbf{p}}_{\mathbf{at}}}{\sigma}_{p_{at}}^2, $$

and

$$ \mathrm{V}\mathrm{a}\mathrm{r}\left({\mathbf{m}}_{\mathbf{at}}\right)={\mathbf{G}}_{{\mathbf{m}}_{\mathbf{at}}}{\sigma}_{m_{at}}^2, $$

where ${\mathbf{G}}_{{\mathbf{p}}_{\mathbf{at}}}$ and ${\mathbf{G}}_{{\mathbf{m}}_{\mathbf{at}}}$ are the genomic relationship matrices of the paternal and maternal gametes, respectively. Let ${\mathbf{M}}_{{\mathbf{p}}_{\mathbf{at}}}$ and ${\mathbf{M}}_{{\mathbf{m}}_{\mathbf{at}}}$ be the *n* × *N*_*snp*_ matrices that specify the coefficients of *a*_*m*_ and *a*_*f*_ in Table 1; then, the elements of ${\mathbf{M}}_{{\mathbf{p}}_{\mathbf{at}}}$ and ${\mathbf{M}}_{{\mathbf{m}}_{\mathbf{at}}}$ for the *i*^*th*^ individual at the *j*^*th*^ SNP are calculated as follows:

$$ {\mathbf{M}}_{{\mathbf{p}}_{\mathbf{at}}\ i,j}\left\{\begin{array}{ll}{q}_j\hfill & \left({A}_1\right)\hfill \\ {}-\left(1-{q}_j\right)\hfill &\ \left({A}_2\right)\hfill \end{array}\right. $$

and

$$ {\mathbf{M}}_{{\mathbf{m}}_{\mathbf{at}}\ i,j}\ \left\{\begin{array}{cc}\hfill {q}_j\hfill & \hfill \left({A}_1\right)\hfill \\ {}\hfill -\left(1-{q}_j\right)\hfill & \hfill \left({A}_2\right)\hfill \end{array}\right.. $$

Therefore, **p**_**at**_ and **m**_**at**_ are as follows:

$$ {\mathbf{p}}_{\mathbf{at}}={\mathbf{M}}_{{\mathbf{p}}_{\mathbf{at}}}{\boldsymbol{\upalpha}}_{\mathbf{m}} $$

and

$$ {\mathbf{m}}_{\mathbf{at}}={\mathbf{M}}_{{\mathbf{m}}_{\mathbf{at}}}{\boldsymbol{\upalpha}}_{\mathbf{f}}, $$

where **α**_**m**_ and **α**_**f**_ are the *N*_*snp*_ -dimensional vectors of *α*_*m*_ and *α*_*f*_, respectively. The variance of **p**_**at**_ is equal to:

$$ \mathrm{V}\mathrm{a}\mathrm{r}\left({\mathbf{p}}_{\mathbf{at}}\right)={\mathbf{M}}_{{\mathbf{p}}_{\mathbf{at}}}{\mathbf{M}}_{{\mathbf{p}}_{\mathbf{at}}}^{\hbox{'}}\mathrm{V}\mathrm{a}\mathrm{r}\left({\alpha}_m\right). $$

The variance of the paternal gametic effect $\left({\sigma}_{p_{at}}^2\right)$ is the sum of the variances of *α*_*m*_ at all SNPs as follows:

$$ {\sigma}_{p_{at}}^2={\displaystyle \sum_j^{N_{snp}}}{q}_j\left(1-{q}_j\right)\mathrm{V}\mathrm{a}\mathrm{r}\left({\alpha}_m\right). $$

From this equation, Var(**p**_**at**_) can be rewritten as follows:

$$ \mathrm{V}\mathrm{a}\mathrm{r}\left({\mathbf{p}}_{\mathbf{at}}\right)=\frac{{\mathbf{M}}_{{\mathbf{p}}_{\mathbf{at}}}{\mathbf{M}}_{{\mathbf{p}}_{\mathbf{at}}}^{\hbox{'}}}{{\displaystyle {\sum}_j^{N_{snp}}}{q}_j\left(1-{q}_j\right)}{\sigma}_{p_{at}}^2. $$

Consequently,

$$ {\mathbf{G}}_{{\mathbf{p}}_{\mathbf{at}}}=\frac{{\mathbf{M}}_{{\mathbf{p}}_{\mathbf{at}}}{\mathbf{M}}_{{\mathbf{p}}_{\mathbf{at}}}^{\hbox{'}}}{{\displaystyle {\sum}_j^{N_{snp}}}{q}_j\left(1-{q}_j\right)}. $$

Similarly,

$$ {\mathbf{G}}_{{\mathbf{m}}_{\mathbf{at}}}=\frac{{\mathbf{M}}_{{\mathbf{m}}_{\mathbf{at}}}{\mathbf{M}}_{{\mathbf{m}}_{\mathbf{at}}}^{\hbox{'}}}{{\displaystyle {\sum}_j^{N_{snp}}}{q}_j\left(1-{q}_j\right)}. $$

### Stochastic simulation

A historical population was simulated to establish mutation-drift equilibrium. The simulated genome comprised 10 chromosomes, each 1 Morgan long, containing 100 000 randomly spaced SNPs and 1000 biallelic quantitative trait loci (QTL). In the first generation of the historical population, the initial allele frequencies of all SNPs and QTL were assumed to be 0.5. A recurrent mutation process was applied with a mutation rate for SNPs and QTL of 1.0 × 10^−4^ per locus per generation. Recombinations were sampled from a Poisson distribution with a mean of 1 per Morgan and then randomly placed along the chromosome. The historical population evolved over 20 000 generations of random selection and random mating, with a population size of 500 (250 males and 250 females) to reach mutation-drift equilibrium [23].

After 20 000 historical generations, the base population (G0) was generated. In G0, the population size decreased to 300 (150 males and 150 females). 10 000 markers and 200 QTL were randomly selected from the segregating SNPs and QTL with minor allele frequencies greater than 0.05. Therefore, *N*_*snp*_ was equal to 10 000. Let *Q*_1_ and *Q*_2_ be two alleles at each QTL. The genotypic values of *Q*_*1*_*Q*_*1*_*, Q*_*1*_*Q*_*2*_*, Q*_*2*_*Q*_*1*_ and *Q*_*2*_*Q*_*2*_, are given by *a, d*_1_*, d*_2_ and -*a*, respectively. The value of *a* was drawn from a gamma distribution with a shape parameter of 0.42 and its sign was drawn at random with equal chance. For QTL with imprinting, the values of *d*_1_ and *d*_2_ were determined as the product of *a* and the degree of imprinting (*τ*). Let *N*_*m*_ and *N*_*f*_ be the number of QTL that are silencing the paternal alleles and maternal alleles. The total number of QTL with imprinting (*N*_*i*_) was 60 (*N*_*m*_ + *N*_*f*_ = *N*_*i*_), which were randomly chosen from the 200 QTL. The total genetic effect (*g*_*j*_) of the *j*^*th*^ animal was calculated by summing all QTL genotypic values, and its variance $\left({\sigma}_g^2\right)$ was calculated from the variance of the genotypic deviations:

$$ \begin{array}{l}{\sigma}_g^2={\displaystyle \sum_{j=1}^{N_{QTL}}}{\left(1-{q}_j\right)}^2{\left\{2{q}_j{a}_j-2\left(1-{q}_j\right){q}_j{\delta}_j\right\}}^2\\ {}+{\displaystyle \sum_{j=1}^{N_{QTL}}}{q}_j\left(1-{q}_j\right){\left[\left(2{q}_j-1\right){a}_j+\left\{1-2{q}_j\left(1-{q}_j\right)\right\}{\delta}_j+{\varepsilon}_j\right]}^2\\ {}+{\displaystyle \sum_{j=1}^{N_{QTL}}}{q}_j\left(1-{q}_j\right){\left[\left(2{q}_j-1\right){a}_j+\left\{1-2{q}_j\left(1-{q}_j\right)\right\}{\delta}_j-{\varepsilon}_j\right]}^2\\ {}+{\displaystyle \sum_{j=1}^{N_{QTL}}}{q_j}^2{\left\{-2\left(1-{q}_j\right){a}_j-2\left(1-{q}_j\right){q}_j{\delta}_j\right\}}^2,\end{array} $$

where *N*_*QTL*_ is the number of QTL. To obtain phenotypic values, an environmental effect was added to the true genetic value, which was sampled from the normal distribution, $\mathrm{N}\left(0,\left(1-{H}^2\right){\sigma}_g^2/{H}^2\right),$ where *H*^2^ is broad-sense heritability; narrow-sense heritability was set to 0.3. The phenotypic variance was finally standardized to be equal to 1.

After G0, the subsequent five generations (G1 to G5) were generated. In G1 to G5, 30 males were selected by BLUP on the basis of estimated breeding values and randomly mated to 150 dams to produce 300 offspring (150 males and 150 females). The reference population with both phenotypes and genotypes comprised 1200 individuals from G1 to G4, and the test population with only genotypes comprised 300 individuals from G5.

The range of *d*_1_ and *d*_2_, the number of QTL with imprinting (*N*_*i*_), and *N*_*snp*_ were varied to investigate their effects on the performance of GBLUP-I. In the base simulation scenario, *τ* = 1.0, *N*_*i*_ = 60, (*N*_*m*_, *N*_*f*_) = (0, 60), *N*_*snp*_ = 10 000, and paternal and maternal alleles were known. In this scenario, only maternal alleles were silenced. Six alternative scenarios were simulated in addition to the base scenario. In scenario 1, *τ* = 0.5, 0.75, and 1.0 to meet the condition that − *a* ≤ *d*_1_, *d*_2_ ≤ *a*. In scenario 2, *N*_*i*_ = 20, 60, and 100. In scenario 3, (*N*_*m*_, *N*_*f*_) = (0, 60), (15, 45), and (30, 30). In scenario 4, *H*^2^*=* 0.1, 0.3, and 0.5. In scenario 5, *N*_*snp*_ = 2000, 10 000, and 50 000. In scenario 6, the paternal and maternal alleles were assumed to be unknown. Parameter settings are outlined in Table 3. Twenty replicates were simulated for each scenario.

**Table 3:** **Parameters for different scenarios**

**Parameter**	**Scenario**						
	**Base**	**1**	**2**	**3**	**4**	**5**	**6**
τ	1.0	0.5, 0.75,1.0	1.0	1.0	1.0	1.0	1.0
*N*_*i*_	60	60	20, 60, 100	60	60	60	60
(*N*_*m*_, *N*_*f*_)	(0, 60)	(0, 60)	(0, 60)	(0, 60)	(0, 60), (15, 45),(30, 30)	(0, 60)	(0, 60)
*H* ^2^	0.3	0.3	0.3	0.1, 0.3,0.5	0.3	0.3	0.3
*N*_*snp*_	10 000	10 000	10 000	10 000	10 000	2000, 10 000, 50 000	10 000
Paternal and maternal alleles	Known	Known	Known	Known	Known	Known	Known, predicted

Six alternative scenarios were simulated in addition to the base scenario: *τ* = degree of imprinting; *N*_*i*_ = number of QTL with imprinting; *N*_*m*_ and *N*_*f*_ = numbers of QTL silencing paternal and maternal alleles; *H* ^2^ = broad-sense heritability; *N*_*snp*_ = number of SNPs.

### Outline of the analysis

In the base scenario, the paternal and maternal alleles were assumed to be known. However, such information is unknown when using real data, because only genotypes are available. In scenario 6, the maternal and paternal origins of specific alleles (phase) were predicted using genotype and pedigree information processed by AlphaImpute software [24]. The phasing accuracy was measured as the correlation between true and predicted alleles by origin.

Here, we estimated variance components and genetic values using GBLUP and two types of GBLUP-I. Variance components were estimated by average information restricted maximum likelihood (AI-REML) [25]. The reference population dataset was used to predict the genetic effects of the genotyped individuals in the test population. The accuracy of the estimated total genetic value (*ρ*) was assessed as the correlation between estimated and true values. The regression coefficients of total genetic value on its estimate (*b*) was calculated to assess unbiasedness.

## Results

Tables 4 and 5 show the estimates of variance components and the predictive abilities of total genetic values with varying values of *τ* and *N*_*i*_ in scenarios 1 and 2. Total genetic variance was underestimated by GBLUP when the degree of imprinting was high. With GBLUP, the estimated total genetic variances were equal to 97.6%, 91.3%, and 82.1% of true variances for *τ* of 0.5, 0.75, and 1.0, respectively, and 99.3%, 82.1%, and 78.2% of true variances for *N*_*i*_ of 20, 50, and 100, respectively. The estimated total genetic variances by GBLUP-I1 and GBLUP-I2 were almost the same as the true variances regardless of the degree of imprinting.

**Table 4:** **Variance component estimates and predictive abilities with varying degrees of imprinting (** ***τ*** **) in scenario 1**

***τ***	**Method**	${\boldsymbol{\sigma}}_{\boldsymbol{\mathsf{g}}}^{\mathbf{2}}$	${\boldsymbol{\sigma}}_{\boldsymbol{e}}^{\mathbf{2}}$	***ρ***	***b***
0.5	True value	0.293	0.698	-	-
	GBLUP	0.286	0.692	0.626	1.026
	GBLUP-I1	0.300	0.679	0.635	1.010
	GBLUP-I2	0.294	0.679	0.570	0.996
0.75	True value	0.290	0.705	-	-
	GBLUP	0.265	0.716	0.581	1.011
	GBLUP-I1	0.295	0.681	0.599	1.000
	GBLUP-I2	0.300	0.680	0.570	0.989
1.0	True value	0.291	0.701	-	-
	GBLUP	0.239	0.742	0.529	0.982
	GBLUP-I1	0.299	0.681	0.570	0.990
	GBLUP-I2	0.297	0.681	0.565	0.995

Values are the mean of 20 replicates; variance components for each source of genetic variation: ${\upsigma}_{\mathit{\mathsf{g}}}^2$ = total genetic variance; ${\sigma}_e^2$ = residual variance; predictive abilities: *ρ* = accuracy of estimated total genetic value; *b* = regression coefficient of total genetic value on its estimate.

**Table 5:** **Variance component estimates and predictive ability with varying numbers of QTL with imprinting (** ***N***_***i***_ **) in scenario 2**

***N***_***i***_	**Method**	${\boldsymbol{\sigma}}_{\boldsymbol{\mathsf{g}}}^{\mathbf{2}}$	${\boldsymbol{\sigma}}_{\boldsymbol{e}}^{\mathbf{2}}$	$\boldsymbol{\rho}$	***b***
20	True value	0.289	0.705	-	-
	GBLUP	0.287	0.696	0.659	1.078
	GBLUP-I1	0.291	0.689	0.660	1.076
	GBLUP-I2	0.286	0.688	0.596	1.071
60	True value	0.291	0.701	-	-
	GBLUP	0.239	0.742	0.529	0.982
	GBLUP-I1	0.299	0.681	0.570	0.990
	GBLUP-I2	0.297	0.681	0.565	0.995
100	True value	0.293	0.698	-	-
	GBLUP	0.229	0.756	0.518	0.975
	GBLUP-I1	0.298	0.683	0.564	0.982
	GBLUP-I2	0.295	0.664	0.575	0.999

Values are the mean of 20 replicates; variance components for each source of genetic variation: ${\upsigma}_{\mathit{\mathsf{g}}}^2$ = total genetic variance; ${\sigma}_e^2$ = residual variance; predictive abilities: *ρ* = accuracy of estimated total genetic value; *b* = regression coefficient of total genetic value on its estimate.

The prediction accuracies, *ρ* obtained with GBLUP-I1 exceeded those obtained with GBLUP by 1.4%, 3.1%, and 7.8% for *τ* of 0.5, 0.75, and 1.0, respectively, and by 0.2%, 7.8%, and 8.2% for *N*_*i*_ of 20, 50, and 100, respectively. Compared to GBLUP-I1, the *ρ* values obtained with GBLUP-I2 were more affected by the degree of imprinting. When *N*_*i*_ was equal to 60 and 100, the *ρ* values obtained with GBLUP-I2 exceeded those obtained with GBLUP by 6.8% and 11.0%; while, when *N*_*i*_ was equal to 20, *ρ* was smaller with GBLUP-I2 than with GBLUP. For all values of *τ* and *N*_*i*_ the *b* values obtained with GBLUP-I1 and GBLUP-I2 were closer to 1 than with GBLUP. In scenario 3, the predictive abilities of GBLUP-I1 were not affected by the values of *N*_*m*_ and *N*_*f*_ whereas the *ρ* values with GBLUP-I2 decreased as the difference between *N*_*m*_ and *N*_*f*_ decreased (Table 6).

**Table 6:** **Variance component estimates and predictive ability with varying numbers of QTL silencing paternal and maternal alleles (** ***N***_***m***_ **and** ***N***_***f***_ **) in scenario 3**

***N***_***m***_	***N***_***f***_	**Method**	${\boldsymbol{\sigma}}_{\boldsymbol{\mathsf{g}}}^{\mathbf{2}}$	${\boldsymbol{\sigma}}_{\boldsymbol{e}}^{\mathbf{2}}$	$\boldsymbol{\rho}$	***b***
0	60	True value	0.291	0.701	-	-
		GBLUP	0.239	0.742	0.529	0.982
		GBLUP-I1	0.299	0.681	0.570	0.990
		GBLUP-I2	0.297	0.681	0.565	0.995
15	45	True value	0.295	0.698	-	-
		GBLUP	0.234	0.739	0.519	0.981
		GBLUP-I1	0.296	0.686	0.569	1.002
		GBLUP-I2	0.298	0.688	0.549	0.998
30	30	True value	0.290	0.704	-	-
		GBLUP	0.234	0.739	0.519	0.981
		GBLUP-I1	0.296	0.686	0.573	1.009
		GBLUP-I2	0.295	0.684	0.538	0.998

Values are the mean of 20 replicates; variance components for each source of genetic variation: ${\upsigma}_{\mathit{\mathsf{g}}}^2$ = total genetic variance; ${\sigma}_e^2$ = residual variance; predictive abilities: *ρ* = accuracy of estimated total genetic value; *b* = regression coefficient of total genetic value on its estimate.

In scenario 4, for all values of *H*^2^ the estimated variance components obtained with GBLUP-I1 and GBLUP-I2 were close to the true values (Table 7). The performance of GBLUP-I1 and GBLUP-I2 increased with increasing values of *H*^2^. With *H*^2^ of 0.1, 0.3, and 0.5, the *ρ* values obtained with GBLUP exceeded those obtained with GBLUP-I1 by 5.7%, 7.8%, and 9.2% and those obtained with GBLUP-I2 by 4.1%, 6.8%, and 7.1%. In scenario 5, the predictive abilities of GBLUP, GBLUP-I1, and GBLUP-I2 decreased when *N*_*snp*_ decreased from 10 000 to 2000 whereas those were unaltered when *N*_*snp*_ increased from 10 000 to 50 000 (Table 8).

**Table 7:** **Variance component estimates and predictive ability with varying broad-sense heritability (** ***H*** ^2^ **) in scenario 4**

***H*** ^2^	**Method**	${\boldsymbol{\sigma}}_{\boldsymbol{\mathsf{g}}}^{\mathbf{2}}$	${\boldsymbol{\sigma}}_{\boldsymbol{e}}^{\mathbf{2}}$	$\boldsymbol{\rho}$	***b***
0.1	True value	0.096	0.902	-	-
	GBLUP	0.078	0.920	0.367	1.021
	GBLUP-I1	0.094	0.885	0.388	0.990
	GBLUP-I2	0.090	0.886	0.382	0.986
0.3	True value	0.291	0.701	-	-
	GBLUP	0.239	0.742	0.529	0.982
	GBLUP-I1	0.299	0.681	0.570	0.990
	GBLUP-I2	0.297	0.681	0.565	0.995
0.5	True value	0.493	0.500	-	-
	GBLUP	0.403	0.597	0.608	1.010
	GBLUP-I1	0.499	0.492	0.664	1.001
	GBLUP-I2	0.498	0.492	0.651	1.000

Values are the mean of 20 replicates; variance components for each source of genetic variation: ${\upsigma}_{\mathit{\mathsf{g}}}^2$ = total genetic variance; ${\sigma}_e^2$ = residual variance; predictive abilities: *ρ* = accuracy of estimated total genetic value; *b* = regression coefficient of total genetic value on its estimate.

**Table 8:** **Variance component estimates and predictive ability with varying numbers of SNPs (** ***N***_***snp***_ **) in scenario 5**

***N***_***snp***_	**Method**	${\boldsymbol{\sigma}}_{\boldsymbol{\mathsf{g}}}^{\mathbf{2}}$	${\boldsymbol{\sigma}}_{\boldsymbol{e}}^{\mathbf{2}}$	$\boldsymbol{\rho}$	***b***
2000	True value	0.290	0.694	-	-
	GBLUP	0.201	0.770	0.481	0.939
	GBLUP-I1	0.263	0.727	0.521	0.946
	GBLUP-I2	0.256	0.733	0.514	0.948
10 000	True value	0.291	0.701	-	-
	GBLUP	0.239	0.742	0.529	0.982
	GBLUP-I1	0.299	0.681	0.570	0.990
	GBLUP-I2	0.297	0.681	0.565	0.995
50 000	True value	0.292	0.693	-	-
	GBLUP	0.242	0.725	0.536	1.072
	GBLUP-I1	0.296	0.687	0.572	1.070
	GBLUP-I2	0.294	0.694	0.566	1.048

Values are the mean of 20 replicates; variance components for each source of genetic variation: ${\upsigma}_{\mathit{\mathsf{g}}}^2$ = total genetic variance; ${\sigma}_e^2$ = residual variance; predictive abilities: *ρ* = accuracy of estimated total genetic value; *b* = regression coefficient of total genetic value on its estimate.

In scenario 6, the phasing accuracy was equal to 0.979. Prediction accuracies with GBLUP-I1 and GBLUP-I2 were 1.7% and 1.2% lower when paternal and maternal alleles were predicted than when paternal and maternal alleles were known (Table 9).

**Table 9:** **Accuracies of estimated genetic values with predicted paternal and maternal alleles in scenario 6**

**Phasing accuracy**	**Method**	${\boldsymbol{\sigma}}_{\boldsymbol{\mathsf{g}}}^{\mathbf{2}}$	${\boldsymbol{\sigma}}_{\boldsymbol{e}}^{\mathbf{2}}$	$\boldsymbol{\rho}$	***b***
1.0	True value	0.291	0.701	-	-
	GBLUP	0.239	0.742	0.529	0.982
	GBLUP-I1	0.299	0.681	0.570	0.990
	GBLUP-I2	0.297	0.681	0.565	0.995
0.979	True value	0.289	0.705	-	-
	GBLUP	0.239	0.742	0.529	0.982
	GBLUP-I1	0.295	0.690	0.560	0.984
	GBLUP-I2	0.294	0.689	0.558	0.991

Values are the mean (standard error) of 20 replicates; variance components for each source of genetic variation: ${\upsigma}_{\mathit{\mathsf{g}}}^2$ = total genetic variance; ${\sigma}_e^2$ = residual variance; predictive abilities: *ρ* = accuracy of estimated total genetic value; *b* = regression coefficient of total genetic value on its estimate.

## Discussion

### Performance of GBLUP-I

We present a new GBLUP method that includes imprinting effects for the prediction of total genetic value. For all scenarios, the performance of GBLUP-I1 to estimate variance components was always better than that of GBLUP. Prediction accuracies with GBLUP-I1 and GBLUP-I2 increased with increasing degree of imprinting and broad-sense heritability. Prediction accuracies with GBLUP-I2 were strongly affected by the degree of imprinting (Tables 4 and 5) and the difference between the values of *N*_*m*_ and *N*_*f*_ (Table 6).

Method GBLUP-I2 assumes that paternal and maternal gametic effects are independent. However, when there is no imprinting, sire and dam are genetically correlated, and thus paternal and maternal gametic effects are not independent [26] and the accuracy by GBLUP-I2 should be reduced. The reduction of accuracy by GBLUP-I2 would be small with a high degree of imprinting because of the low correlation between paternal and maternal gametic effects. Thus, the performance of GBLUP-I2 increases as the degree of the imprinting and the difference in genetic values between paternal and maternal gametes increase. Meanwhile, when the degree of imprinting is low and there is little difference in the genetic values between paternal and maternal gametes, GBLUP-I1 is preferred for genomic evaluation.

In a previous study with bovine data, when the number of SNPs was greater than 50 000, reliabilities of genomic evaluations remained almost unaltered as the number of SNPs increased [27]. In this study, with a *N*_*snp*_ of 10 000, the average distance between neighboring SNPs was 0.1 cM, which is similar to the distance between SNPs in a bovine dataset that includes 50 000 SNPs. In scenario 5, when *N*_*snp*_ was greater than 10 000, the performances of GBLUP and both GBLUP-I were not affected by various values of *N*_*snp*_. This suggests that high-density and costly chips with more (777 000) SNPs may not be necessary for genomic evaluation, even when imprinting effects exist.

In scenario 6, prediction accuracies obtained with GBLUP-I1 and GBLUP-I2 were higher than those obtained with GBLUP when paternal and maternal alleles were predicted. Thus, both GBLUP-I methods can be applied to real livestock data. The phasing accuracy was improved by increasing sample size [28], number of SNPs [29], and number of high-density genotyped relatives of the individuals to be imputed [24,30], which suggests that the performance of GBLUP-I can be further improved in real livestock data.

### Degree of imprinting and number of QTL with imprinting

GBLUP-I1 and GBLUP-I2 could accurately capture the total genetic variance, whereas GBLUP underestimated the total genetic variance. The difference in estimated total genetic variance between GBLUP-I and GBLUP is caused by the imprinting effect. Here, we calculated imprinting variance as the difference in the estimated total genetic variance between GBLUP-I1 and GBLUP. When *τ* varied from 0.5 to 1.0 and *N*_*i*_ from 20 to 100, imprinting variances were equal to 1.4% to 6.0% and 0.4% to 6.9% of the phenotypic variances (1.0), respectively.

de Vries et al. [31] were the first to estimate imprinting variance in livestock and found that approximately 5% and 4% of the phenotypic variance in back fat thickness and growth rate, respectively, were due to imprinting. More recently, imprinting variances were found to range from 5 to 19% of the total genetic variance for 19 pig performance traits [13] and, on average, to be equal to 28% of the total genetic variance for ultrasonic measurements of body composition in Australian beef cattle [26]. The degree of imprinting reported in our study is similar to those reported in the literature [13,26,31].

### Effects of QTL parameters

Setting QTL parameters may affect the accuracy of genomic predictions. We investigated the effects of the number of QTL, the distribution of their effects, and their location. The values of *N*_*QTL*_ ranged from 50 (*N*_*i*_ = 15) to 1000 (*N*_*i*_ = 300) and QTL were evenly spaced throughout the genome. The value of *a* was drawn from a normal distribution. In these conditions, the accuracies obtained by GBLUP, GBLUP-I1, and GBLUP-I2 were the almost the same as in the base scenario (Table 10).

**Table 10:** **Accuracies of estimated genetic values with varying numbers of QTL, distributions of homozygous genotypic value, and locations of QTL**

**Number of QTL**	**Distribution of homozygous genotypic value (** ***a*** **)**	**QTL location**	**Method**	${\boldsymbol{\sigma}}_{\boldsymbol{\mathsf{g}}}^{\mathbf{2}}$	${\boldsymbol{\sigma}}_{\boldsymbol{e}}^{\mathbf{2}}$	$\boldsymbol{\rho}$	***b***
50	Gamma	Random	GBLUP	0.238	0.740	0.528	0.983
			GBLUP-I1	0.295	0.683	0.565	0.989
			GBLUP-I2	0.296	0.685	0.564	0.992
200	Gamma	Random	GBLUP	0.239	0.742	0.529	0.982
			GBLUP-I1	0.299	0.681	0.570	0.990
			GBLUP-I2	0.297	0.681	0.565	0.995
1000	Gamma	Random	GBLUP	0.235	0.745	0.527	0.979
			GBLUP-I1	0.297	0.686	0.568	0.994
			GBLUP-I2	0.294	0.684	0.563	0.993
200	Normal	Random	GBLUP	0.239	0.741	0.529	0.980
			GBLUP-I1	0.294	0.681	0.566	0.991
			GBLUP-I2	0.295	0.683	0.560	0.994
200	Gamma	Evenly spaced	GBLUP	0.245	0.738	0.531	0.989
			GBLUP-I1	0.295	0.688	0.572	0.997
			GBLUP-I2	0.294	0.689	0.567	0.999

Values are the means of 20 replicates; variance components for each source of genetic variation: ${\sigma}_g^2$ = total genetic variance; ${\sigma}_e^2$ = residual variance. Predictive abilities: *ρ* = accuracy of estimated total genetic value; *b* = regression coefficient of total genetic value on its estimate.

### Significance of genetic effects in GBLUP-I

This study partitioned the total genetic value into three estimated genetic effects (*α*, *δ*, and *ε* terms) in GBLUP-I1. However, there is no biological meaning for these genetic effects. In order to estimate a breeding value and a dominance deviation, the genetic values should be defined by sex, as presented by Spencer [19]. In such a model, the number of variance components would be doubled and the covariance between the breeding value and dominance deviation would not be equal to 0. These factors would collectively reduce the accuracy of genetic evaluations.

### Practical use of GBLUP-I

When a dominance effect exists, assortative mating or mate allocation can boost the field performance of livestock [32,33]. Similarly, when an imprinting effect exists, the performance of livestock can be improved by optimizing matings, because the genotypic values of *A*_*1*_*A*_*2*_ and *A*_*2*_*A*_*1*_ can be distinguished and evaluated accurately. Let *pr*_*ij*_ (A_1_A_1_) *pr*_*ij*_ (*A*_*1*_*A*_*2*_) *pr*_*ij*_ (*A*_*2*_*A*_*1*_) and *pr*_*ij*_(*A*_*2*_*A*_*2*_) be the probabilities of the genotypes *A*_*1*_*A*_*1*_*, A*_*1*_*A*_*2*_*, A*_*2*_*A*_*1*_*,* and *A*_*2*_*A*_*2*_ for the *i*^*th*^ offspring of future matings and the *j*^*th*^ marker. In GBLUP-I1, the elements of coefficient matrices for these offspring (i.e., **M**_**a**_**, M**_**d**_, and **M**_**i**_ ) can be calculated from the products of coefficients and the genotype probabilities. For example, the element of **M**_**i**_ for offspring is as follows:

$$ {\mathbf{M}}_{\mathbf{i}\ i,j} = \left\{\begin{array}{l}0\ \left({A}_1{A}_1\right)\hfill \\ {}\ 1\times p{r}_{ij}\left({A}_1{A}_2\right)\ \left({A}_1{A}_2\right)\hfill \\ {}-1 \times p{r}_{ij}\left({A}_2{A}_1\right)\ \left({A}_2{A}_1\right)\hfill \\ {}\ 0\ \left({A}_2{A}_2\right)\hfill \end{array}\right.. $$

Likewise, in GBLUP-I2, the elements of ${\mathbf{M}}_{{\mathbf{p}}_{\mathbf{at}}}$ and ${\mathbf{M}}_{{\mathbf{m}}_{\mathbf{at}}}$ for the offspring of future matings can be calculated. Thus, the total genetic effects for the offspring of future matings can be predicted and maximized by using an optimum mating plan.

## Conclusions

This study proposed two GBLUP methods i.e., GBLUP-I1 and GBLUP-I2, which include imprinting effects at the genotypic and gametic levels, respectively. The GBLUP-I1 and GBLUP-I2 methods accurately estimated the variance components and improved unbiasedness regardless of parameter settings. The accuracies of estimated total genetic values in GBLUP-I1 and GBLUP-I2 increased with increasing degree of imprinting and broad-sense heritability. Compared to GBLUP, the accuracies of estimated total genetic values obtained with GBLUP-I1 were always higher. Thus, in general, GBLUP-I1 should be applied for genetic evaluation. However, GBLUP-I2 is preferred when the imprinting effect is large and the genetic effects differ substantially between paternal and maternal gametes. After predicting the total genetic value by both GBLUP-I methods, assortative mating or mate allocation could be used to boost the field performance of livestock.
